# Synergetic Effect of Ultrasound, the Heterogeneous Fenton Reaction and Photocatalysis by TiO_2_ Loaded on Nickel Foam on the Degradation of Pollutants

**DOI:** 10.3390/ma9060457

**Published:** 2016-06-08

**Authors:** Shan Qiu, Shanwen Xu, Guangming Li, Jixian Yang

**Affiliations:** 1 School of Municipal and Environmental Engineering, Harbin Institute of Technology, Harbin 150006, China; xsw1978828@126.com (S.X.); liguangming@hit.edu.cn (G.L.); yangxj@hit.edu.cn (J.Y.); 2 State Laboratory of Urban Water Resources and Environment, Harbin Institute of Technology, Harbin 150006, China

**Keywords:** Fenton reaction, nickel foam, TiO_2_, photocatalysis, acetochlor

## Abstract

The synergistic effect of ultrasound, the heterogeneous Fenton reaction and photocatalysis was studied using a nickel foam (NF)-supporting TiO_2_ system and rhodamine B (RhB) as a target. The NF-supporting TiO_2_ system was prepared by depositing TiO_2_ on the skeleton of NF repeatedly and then calcining it. To optimize the conditions and parameters, the catalytic activity was tested in four systems (ultrasound alone (US), nickel foam (NF), US/NF and NF/US/H_2_O_2_). The optimal conditions were fixed at 0.1 g/mL NF, initial 5.00 mg/L RhB, 300 W ultrasonic power, pH = 3 and 5.00 mg/L H_2_O_2_. The effects of the dissolution of nickel from NF and quenching of the Fenton reaction were studied on degradation efficiency. When the heterogeneous Fenton reaction is combined with TiO_2_-photocatalysis, the pollutant removal efficiency is enhanced significantly. Through this synergistic effect, 22% and 80% acetochlor was degraded within 10 min and 80 min, respectively.

## 1. Introduction

As one of advanced oxidation processes (AOP), the Fenton reaction can efficiently degrade various pollutants [1,2,3]. Free radicals are produced in the Fenton reaction, which can damage and degrade organic molecules. Hydroxyl radicals (OH•) are the main product of the Fenton reaction with high reactivity. They convert organic molecules into CO_2_, H_2_O and other inorganic ions [4,5]. The other advantage of the Fenton reaction is its low cost. The Fenton reaction can stimulate H_2_O_2_ without extra energy, reactions, sophisticated equipment or pressurization devices. The whole process is easy to operate and control. The oxidation in the Fenton reaction can be used in all kinds of waste water treatment, included in phenol wastewater, agricultural wastewater, coking wastewater, cyanide wastewater, dye wastewater, dye intermediate wastewater and landfill leachate. Therefore, the Fenton reaction is a kind of AOP that potentially can be used in environmental pollutant treatment areas [6,7,8].

The homogeneous Fenton system has some limitations, such as a narrow range of pH (2–4) and the production of much sludge after the Fenton process. At present, the heterogeneous Fenton reaction has become the focus of research. To overcome these drawbacks of the homogeneous Fenton system, the heterogeneous Fenton system was prepared by loading catalysts on the carrier. The catalysts included Fe_3_O_4_, TiO_2_, Fe_2_O_3_, and so on. The carriers have been reported, such as activated carbon Al_2_O_3_, mesoporous silica, zeolites and clay mineral carbon aerogel [9,10].

Among these supports, nickel foam (NF) has been widely investigated in various energy-storage devices, such as supercapacitors, full cells and batteries [11,12,13,14], owing to its distinguished advantages, such as a distinct three-dimensional porous structure, a big surface area, high conductivity, good structural stability, ease of processing, low cost, and so on. NF was found as the most suitable material for the generation of H_2_O_2_. The NF was also found to be a suitable cathode material for the electro-Fenton treatment of recalcitrant compounds in continuous mode [12,14].

The photocatalysis process produced a positive hole (h^+^) in the valence band and an electron (e^−^) in the conduction band. The positive hole oxidizes either the organic pollutant or H_2_O_2_ to induce hydroxyl radicals. When the Fenton reaction is combined with TiO_2_-photocatalysis, the pollutant removal efficiency is expected to be enhanced [15,16,17]. In addition, it has also been reported that ultrasound is most commonly used for waste treatment, which can degrade pollutants [18].

In the present work, a composite catalyst was prepared by TiO_2_ deposited on NF. We combined the Fenton reaction with TiO_2_-photocatalysis by a composite of TiO_2_ carried by NF. The synergistic effect of the Fenton reaction and photocatalysis was studied for four Fenton systems using rhodamine B (RhB) as a target. The degradation efficiency of acetochlor under optimum reaction conditions was measured.

## 2. Materials and Methods

### 2.1. Materials

RhB, 30% H_2_O_2_ and other routine chemicals were purchased from Shenshi Chem (Wuhan, China). The NF sheet was purchased from Suzhou Jiashide foam Pioneer Metals Corporation (Suzhou, China).

### 2.2. Preparation of TiO_2_ Loaded on NF

An NF was used as the carrier of TiO_2_. The disk was a piece of foam (30 mm in diameter and 10 mm in length) with about 30 cells per linear inch (CPI). The porosity, aperture and bulk density were 70%, 0.4 mm and 0.5 g/cm^3^, respectively.

The NF was soaked in an aqueous slurry of TiO_2_ sol-gel. In a typical procedure, 150 mL tetrabutyl titanate were added into 180 mL water drop-wisely. Then, it was dried at room temperature overnight. The sample was washed and dried. This procedure was repeated until the TiO_2_ coating reached up to 5 wt %. Then, the sample was calcined at 400 °C for 2 h in a N_2_ atmosphere. The loading weight was calculated according to the weight of coated and uncoated foam.

The structure and morphology of the sample were observed by environmental scanning electron microscopy (ESEM, Quanta FEG 450, FEI Company, Hillsboro, OR, USA) together with a backscattered electron (BSE, FEI Company, Hillsboro, OR, USA).

### 2.3. Catalytic Activity Testing

To obtain the optimal conditions and parameters, two series of systems were designed. One is the catalytic activity tests of four systems (ultrasound alone (US), nickel foam (NF), foam nickel with ultrasound (US/NF) and the heterogeneous system (NF/US/H_2_O_2_)). After the optimal conditions were obtained, the other series of systems were conducted for the RhB degradation experiment (NF/TiO_2_ + H_2_O_2_ in the dark, US + NF/TiO_2_ + H_2_O_2_ in the dark, UV + NF/TiO_2_ + H_2_O_2_, UV + US + NF/TiO_2_ + H_2_O_2_). NF was fixed at 0.1 g/mL in the experiment.

RhB (5 mg/L) degradation was used to evaluate the efficiency of catalysis. The maximum absorption wavelength was 554 nm. A linear relationship was built between RhB concentration and absorbance. The concentration of RhB can be determined by the absorbance by a UV-VIS spectrophotometer (724, Shanghai 3rd Analytical Instrument Ltd., Shanghai, China). The reaction temperature was maintained at 25 ± 1 °C. The pH values were adjusted to the required value by 0.1 mg/L HCl and 0.1 mol/L NaOH. During the experiment, an ultrasonic processor with a frequency of 20 kHZ (FS-600N, Shanghai Shengxi, Shanghai, China) was used with the ultrasonic probe at 1.5 cm below the water surface. For the UV light, a mercury lamp (150 W, wavelength 365 nm, FoShan Lighting, Foshan, China) was used as the light source, and the distance between the lamp and the reaction liquid was 15 cm. The irradiance reaching the sample was about 0.1 W/cm^2^.

#### 2.3.1. Analysis of Ni^2+^

An inductively-coupled plasma mass spectrometer (ICP-MS) (Agilent 7500a, Palo Alto, CA, USA) was used to determine the amount of Ni^2+^. The supernatant obtained from the above experiment was analyzed for metal content by ICP-MS in order to evaluate the effect of Ni^2+^.

#### 2.3.2. Quenching of Catalysis

Quenching tests were carried out to determine the active radicals. Tertiary butanol was used as the quenching reagent with different concentrations.

#### 2.3.3. Degradation of Acetochlor

A known amount of acetochlor (5.0 mg·L^−1^) was treated by a combination of the heterogeneous Fenton reaction and photocatalysis by a composite of TiO_2_ carried by NF. The acetochlor concentration was measured in real time by a gas chromatograph-mass spectrometer (GC/MS, Palo Alto, CA, USA) [19].

#### 2.3.4. Measurement of Hydroxyl Radical

One milliliter of the reaction solution was mixed with 2 mL of 0.2 mol/L dimethyl sulfoxide and 2 mL of 15 mmol/L 4-Benzoylamino-2,5-dimethoxybenzenediazonium chloride hemi(zinc chloride) salt for 10 min in the dark reaction. One milliliter of pyridine and 3 mL of extract solution (toluene/butanol: 1/3) were added and fully mixed. The toluene/butanol phase was taken out for measurement of absorbance at 420 nm.

## 3. Results and Discussion

### 3.1. Removal of RhB under Different Fenton Reaction Systems

For rapid screening of the AOPs, a dye (RhB) was used as a target to study the removal of RhB under different Fenton catalysis systems [20,21]. Four AOP systems were compared to each other: US alone, NF alone, US/NF, NF and US/NF/H_2_O_2_ for the removal efficiency of RhB. The solution contained 0.1 g/mL NF. The initial RhB concentration was 5.00 mg/L with a pH value of three. The initial concentration of H_2_O_2_ is 5.0 mmol/L, and the ultrasonic power is 300 W. The removal efficiency of RhB was calculated after 25 min of reaction time.

(1) $$\text{Removal Efficiency}=\frac{\text{terminal concentration}}{\text{initial concentration}}\times 100\%$$

**Figure 1:**
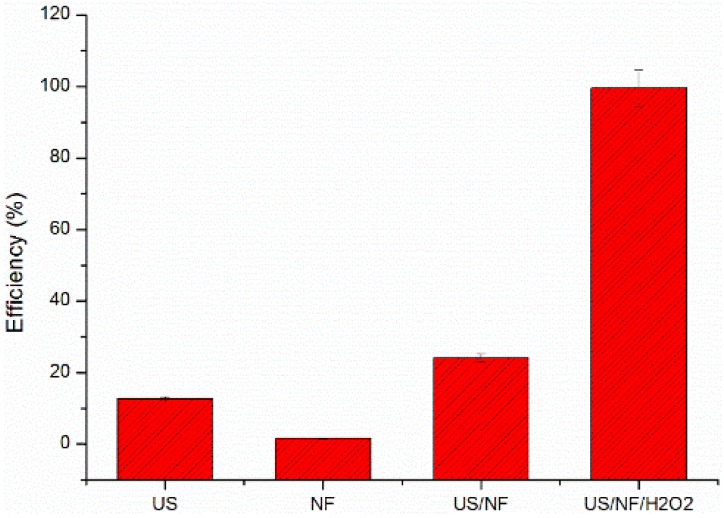
Removal efficiency of rhodamine B (RhB) in different oxidation processes. US, ultrasound; NF, nickel foam.

**Figure 2:**
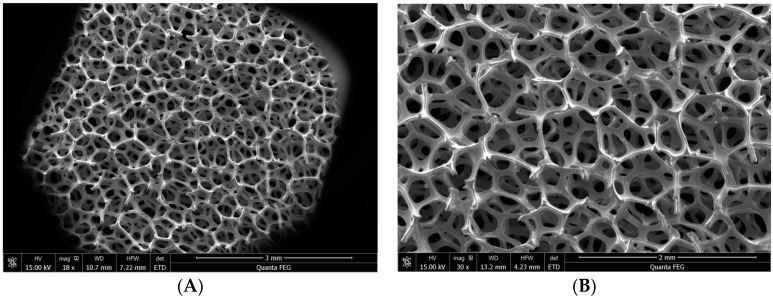
SEM images of nickel foam with different magnification (**A**: 1800×; **B**: 3000×).

Figure 1 shows the removal efficiency of RhB under different Fenton catalytic systems. Under 300-W ultrasonic radiations alone, the removal rate of RhB is about 12.68%, indicating that ultrasonic energy is inefficient at degrading RhB. In the presence of NF alone, the removal rate of RhB was 1.56%. In this case, the removal of RhB may be attributed to the absorption of RhB on the surface of NF, as shown in Figure 2. Combining US with NF, the removal efficiency was improved significantly. The removal rate of RhB increased to 24.13%. For the Fenton catalytic system in the presence of H_2_O_2_ with US and NF, the RhB removal rate can reach 99.53%, far higher than that of other groups.

As shown in Figure 2, NF has the three-dimensional network structure, with a high porosity and a large specific surface area. Specifically, NF was characterized with high permeability and good mechanical properties [22]. Therefore, NF can provide more cavitation nuclei and strengthen the ultrasonic cavitation effect. Consequently, NF increased the number of free radicals. Besides, due to adsorption-desorption in the gas-liquid boundary layer, the presence of NF increased the gas content in the solution, also strengthening the ultrasonic cavitation effect [23,24,25]. Under US, H_2_O_2_ could produce OH• as the following Equations (2) and (3).

(2) $$H_{2}O_{2}+\text{ultrasonic energy }\to \text{ OH}•$$

(3) $$H_{2}O_{2}+\text{ultrasonic energy }\overset{\text{NF}}{\underset{\text{enhancing}}{\to }}\text{ OH}•$$

In the presence of H_2_O_2_, free radicals in solution increased obviously. The order of RhB removal efficiency ranked as US/NF/H_2_O_2_ > US/NF > US > NF.

### 3.2. Effect of Ultrasonic Power on Degradation of RhB

**Figure 3:**
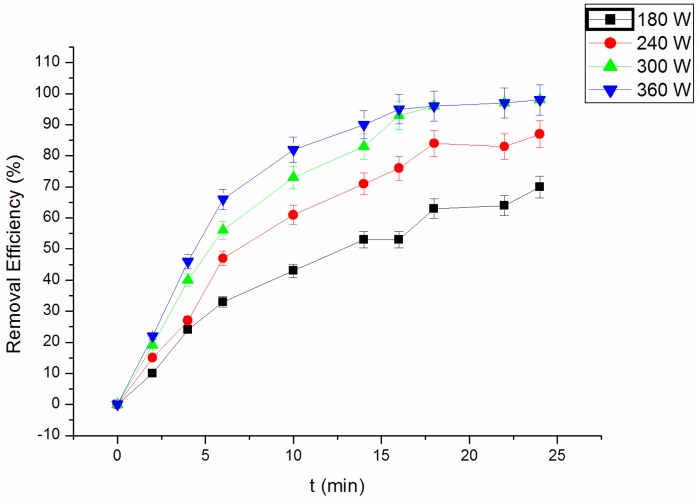
Effect of ultrasonic power on the RhB removal efficiency.

Figure 3 showed the ultrasonic power’s influence on the removal efficiency. An experiment was conducted under the conditions 0.1 g/mL NF, initial 5.00 mg/L RhB, the initial pH of three and 5.00 mg/L H_2_O_2_. The ultrasonic power was set as 180, 240, 300 and 360 W, respectively. The result shows that RhB removal efficiency increases with the increase of ultrasonic power. After 25 min of reaction, the removal rates of RhB were 70.12%, 87.22%, 99.53% and 98.09%, respectively. With ultrasonic power enhanced, it is helpful to produce turbulence in the solution and to enhance the degree of cavitation, which favors the degradation of RhB. However, when the ultrasonic power is too high, the cavitation bubbles become so big as to form a barrier, reducing the application of the ultrasonic energy and decreasing the catalytic efficiency [26].

When the ultrasonic power increased from 300 to 360 W, the removal efficiency of RhB did not change significantly. With the consideration of the energy consumption, the ultrasonic power can be fixed at 300 W.

### 3.3. Effect of H_2_O_2_ Concentration on the Degradation of RhB

**Figure 4:**
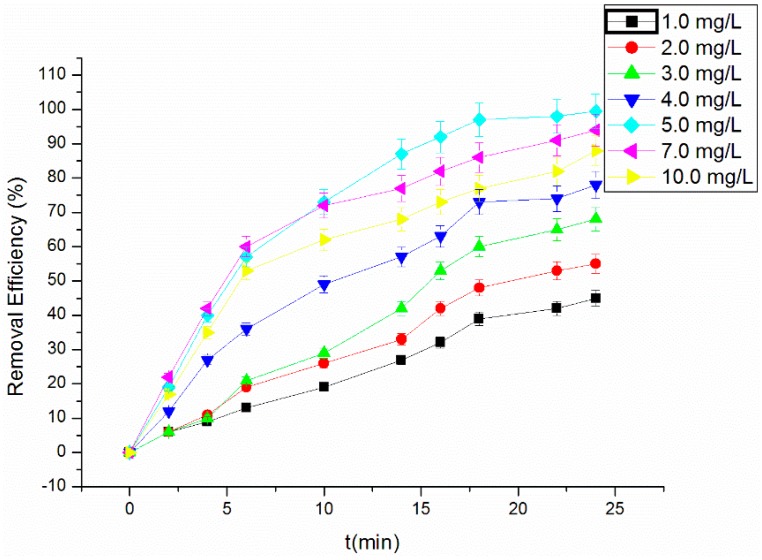
Effect of H_2_O_2_ dosage on the RhB removal efficiency.

The effect of the H_2_O_2_ dosage on the removal efficiency of RhB is shown in Figure 4. The experiment was conducted under the conditions of 0.1 g/mL NF, initial 5.00 mg/L RhB, the initial pH of three and 300 W US. The concentrations of H_2_O_2_ were set as 1.0, 2.0, 3.0, 4.0, 5.0, 7.0 and 10 mg/L, respectively. It can be seen that the removal efficiency of RhB increased along with the concentration of H_2_O_2_. After 25 min of the reaction, the removal efficiency increased from 68.34% to 99.53% in the presence of 5.00 mg/L H_2_O_2_. When the H_2_O_2_ concentration was greater than 5.0 mg/L, however, the removal efficiency decreased slightly. When the H_2_O_2_ concentration was low, with the increase of concentration, US promotes the production of OH• by H_2_O_2_. However, when the H_2_O_2_ concentration is too high, the reaction may produce OH• inhibitors and reduce the discharge of OH• as the following Equations (4) and (5). The concentrations of H_2_O_2_ can be fixed at 5.00 mg/L.

H_2_O_2_ +·OH• → HO_2_• + H_2_O (4)

HO_2_• +·OH• → H_2_O + O_2_ (5)

### 3.4. Effect of Initial pH Value on the RhB Removal Efficiency

**Figure 5:**
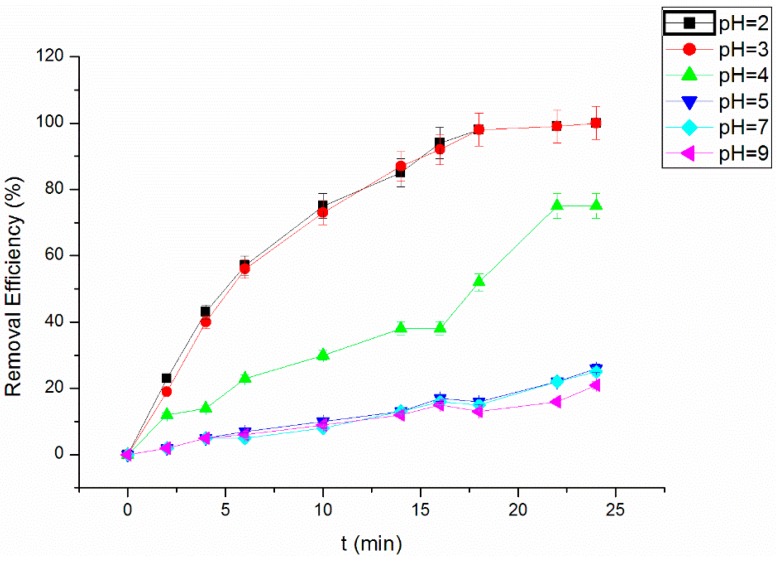
Effect of the initial pH value on the RhB removal efficiency.

Figure 5 shows the initial pH value’s influence on the removal efficiency. The experiment was conducted under the conditions of 0.1 g/mL NF, initial 5.00 mg/L RhB, 300-W ultrasonic power and 5.00 mg/L H_2_O_2_. The initial pH value was adjusted to 2, 3, 4, 5, 7 and 9, respectively. The result shows that with pH 2–3, the RhB removal effect is highest. When the pH increases to four, the removal rate of RhB was significantly reduced. The pH value influenced the form of RhB in solution. With lower pH values, RhB can enter the cavitation bubbles mainly in molecular form, and directly react with the free radicals produced by the cavitation. When the pH value is higher, RhB mainly exists in ionic form, which can quench the free radical reaction, so the removal rate decreased. The optimum conditions were fixed as 5.00 mg/L RhB, pH 3, 300-W ultrasonic power and 5.00 mg/L H_2_O_2_.

### 3.5. Dissolution of Nickel from NF

The investigation of nickel dissociation is of great significance to the Fenton reaction. The dissolution of nickel ions may produce homogeneous/heterogeneous mixing system, rather than a complete heterogeneous Fenton system. Nickel ions in NF may cause secondary pollution of heavy metal [27]. The dissolution may damage the structure of NF and decreases its catalytic activity. In this study, after 25 min of the Fenton reaction under the optimum conditions, the concentration of nickel ion was measured as 2.50 mg/L by ICP-MS.

**Figure 6:**
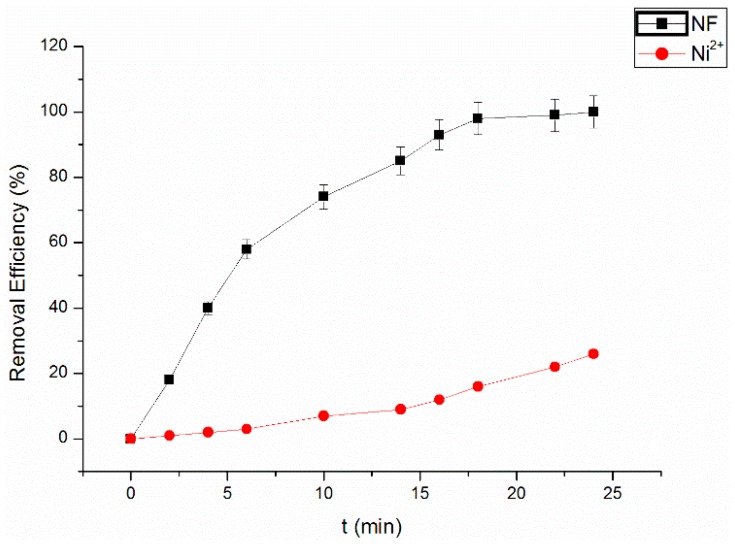
Comparison of Fenton catalysis with Ni^2+^ alone for removal efficiency.

To evaluate the role of nickel ions in the Fenton reaction, the experiment was conducted in the presence of 2.50 mg/L Ni^2+^ without NF under the same optimum conditions. The results are shown in Figure 6, suggesting that after 25 min of reaction, the removal efficiency of RhB was 18.26%. In the presence of NF, the RhB removal efficiency can reach more than 99%, almost completely degrading the RhB. The removal of RhB can be attributed to the heterogeneous Fenton reaction and the enhanced cavitation effect on the surface of the NF.

### 3.6. Quenching of the Fenton Reaction

**Figure 7:**
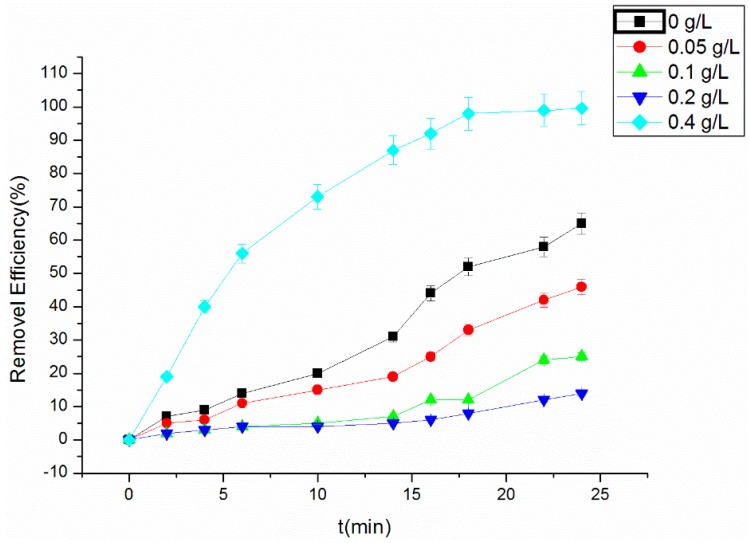
Effect of tertiary butanol on the RhB removal efficiency.

Figure 7 shows the removal of RhB under the optimal conditions in the presence of tertiary butanol concentration. The results suggested that the removal efficiency of RhB decreased significantly with the increase of the quencher tertiary butanol. With a tertiary butanol concentration of 0.4 g/L, the removal efficiency of RhB in the reaction system is less than 3%. In the process of the reaction, the quencher can capture the free radical OH• prior to the reaction of RhB with OH•, producing a highly selective and inert intermediate [11,28], thus hindering the generation of OH• and the degradation of RhB. The absorbance of RhB in the presence of tertiary butanol was also measured, and the result showed that tertiary butanol did not influence RhB adsorption.

### 3.7. The Synergetic Effect of the Fenton Reaction and Photocatalysis

**Figure 8:**
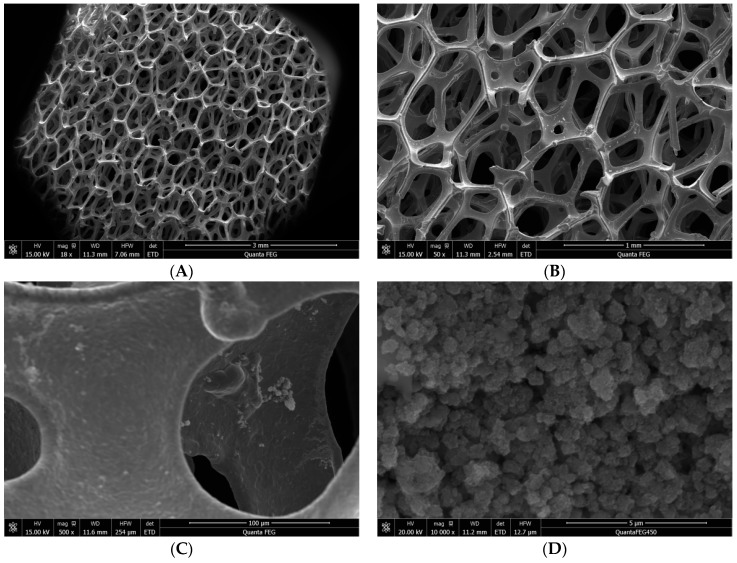

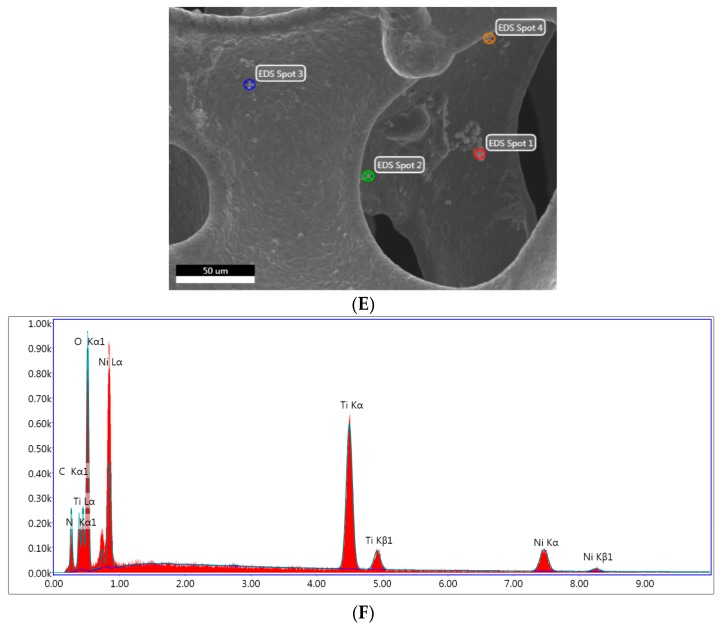
The morphology of TiO_2_ deposited on nickel foam samples (**A**–**D**) and the EDX spectrum (**E**,**F**).

The morphology of TiO_2_ deposited on NF samples was investigated by FESEM, as is shown in Figure 8A–D. It can be seen that TiO_2_ was deposited on the skeleton of NF. TiO_2_ particles spread uniformly without aggregation, as shown in Figure 8D. Five areas on the skeleton of NF were selected to analyze the element by FSEM-EDX, which is shown in Figure 8E,F. The results proved that TiO_2_ was modified on the skeleton successfully. The average percentage of Ti element is about 23% in Figure 8F. This is enough to perform the photocatalytic activity. The application of NF monoliths in catalytic reactors favors the heat and mass transfer. Specifically, the permeability of the NFs to solution can be related to macroscopic properties, such as the number of pores per unit length, the apparent density or the void fraction [29,30,31].

**Figure 9:**
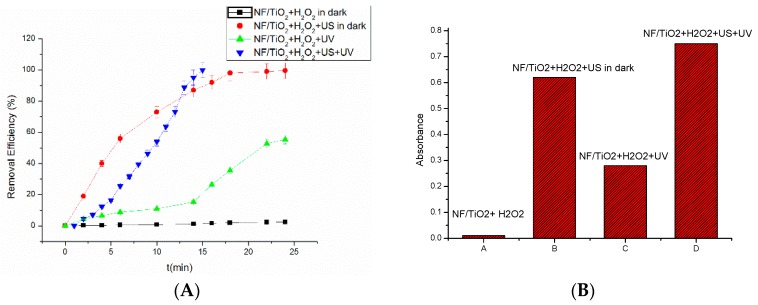
RhB removal efficiency by different systems (**A**) and the level of OH• radicals for different systems (**B**).

Comparative experiments of RhB degradation were conducted to test the catalytic activity of the four catalytic systems. They are NF/TiO_2_ + H_2_O_2_ in the dark, NF/TiO_2_ + H_2_O_2_ with US in the dark, NF/TiO_2_ + H_2_O_2_ with UV and NF/TiO_2_ + H_2_O_2_ with US and UV. The degradation of 5 mg/L of RhB as a function of the reaction time is shown in Figure 3. As shown in Figure 9, it was seen that approximately 2.44% of RhB was degraded in 25 min for the case of NF/TiO_2_ + H_2_O_2_ in the dark, which is similar to NF alone. The degradation rate reached 99.3% using NF/TiO_2_ + H_2_O_2_ with US in the dark and 55.4% NF/TiO_2_ + H_2_O_2_ with UV, which were both much higher than NF/TiO_2_ + H_2_O_2_ in the dark. As for NF/TiO_2_ + H_2_O_2_ with US and UV, RhB was degraded completely within 15 min. It was revealed that the combined effect of the Fenton reaction and photocatalysis enhanced the removal of RhB significantly.

Therefore, the combined Fenton/photocatalysis process showed a synergistic effect in the combination of US, the Fenton reaction and photocatalysis.

The role of NF, TiO_2_ and H_2_O_2_ in the combined sulfate radical based Fenton reaction/photocatalysis system was analyzed. Titanium dioxide, particularly in the anatase form, is a photocatalyst under ultraviolet (UV) light. TiO_2_ can produce electrons (e^−^) and holes (h^+^). The strong oxidative potential of the positive holes oxidizes water to create hydroxyl radicals. It can also oxidize organic molecules directly as the following Equations (6)–(8).

(6) $$\text{hv}+{\text{TiO}}_{2}\;\to {\text{ hole}}^{+}+{\text{electron}}^{-}$$

(7) $${\text{electron}}^{-}+H_{2}O_{2}\;\to \;{\text{OH}}^{-}+\text{OH}•$$

(8) $${\text{hole}}^{+}+H_{2}O_{2}\;\to \;H^{+}+{\text{HO}}_{2}•$$

Both the Fenton reaction and photocatalysis can produce OH• to degrade organic material. It is easier to produce the hydroxyl radical in the presence of H_2_O_2_ by electrons (e^−^) and holes (h^+^). The cavitation effect also favors the separation of e^−^ and h^+^, which is available for oxidation of adsorbed organic compounds. The cavitation effect also facilitates the generation of OH• by adsorbed water molecules [17,18]. Theron’s group reported the synergistic effect of photocatalysis and ultrasound on degrading pollutants [32]. They believe that hydrogen peroxide was produced by ultrasound in the first stage. Hydrogen peroxide is then rapidly degraded to radicals at the TiO_2_ surface under irradiation. In our study, both TiO_2_ photocatalysis and Fenton catalysis can produce OH•. The amount of OH• of NF/TiO_2_ + H_2_O_2_ with US and UV is expected to maintain a higher level than the other systems. In the measurement, dimethyl sulfoxide was used as a molecular probe of OH• radical in the aqueous phase to determine the yield of OH• radical by a spectrophotometer. NF/TiO_2_ + H_2_O_2_ almost did not yield OH• radicals, while NF/TiO_2_ + H_2_O_2_ with US and UV yielded the highest level of OH• radicals. The OH• level of NF/TiO_2_ + H_2_O_2_ with UV is lower than NF/TiO_2_ + H_2_O_2_ with US in the dark, suggesting that the effect of US is more obvious than UV.

The process of ultrasonic propagation in the medium induced a variety of physical and chemical effects, including a mechanical effect, a thermal effect, cavitation and a mass transfer effect. Ultrasonic cavitation caused by instantaneous high temperature and high pressure decomposed water and also produced OH• free radicals. In addition, the production and collapse of cavitation bubbles favors the mass transfer and diffusion of free radicals into the whole solution. The occurrence of chemical transformations occurring in and/or on the cavitation bubbles has been reported [33]. Besides, the high temperature caused by ultrasonic cavitation and the fast diffusion of substance also accelerate the degradation rate.

Therefore, we believed that H_2_O_2_ and the cavitation effect also enhanced the photocatalytic performance by inhibiting photo-excited electron–hole pair recombination. As for the hydroxyl radical produced by US, it is more stable due to the presence of the strong oxidative potential of the electrons e^−^ and holes h^+^.

### 3.8. Degradation of Acetochlor by the Synergetic Effect

**Figure 10:**
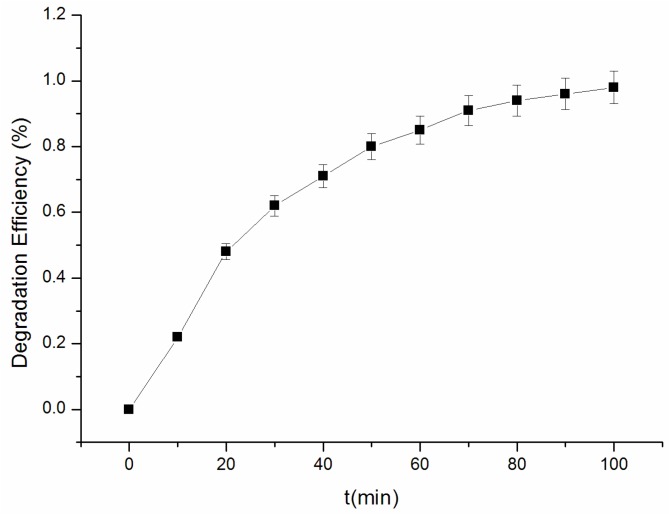
Degradation of acetochlor by the synergetic effect of the Fenton-like reaction and photocatalysis.

As an important herbicide, acetochlor is used widely for the control of annual grasses. However, the problem was raised concerning the environmental contamination, because acetochlor is a probable human carcinogen. Acetochlor can be degraded after treatment. In this study, the degradation efficiency was measured for evaluating the synergetic effect of the Fenton reaction and photocatalysis. Figure 10 shows the concentration decrease with time in the Fenton reaction. The first-order kinetics was observed in the process for the degradation of acetochlor. 22% and 80% acetochlor was degraded within 10 and 80 min, respectively. The degradation efficiency reaches 98% at 100 min. The degradation of acetochlor is very slow by other methods reported in the literature [34,35]. The degradation efficiency ranges from 10% to 50% after several hours.

## 4. Conclusions

In this study, the synergistic effect of the heterogeneous Fenton reaction and photocatalysis was studied using an NF-supporting TiO_2_ system and RhB as a target. The NF-supporting TiO_2_ system was prepared by depositing TiO_2_ on the skeleton of NF repeatedly and then calcining it. To optimize the conditions and parameters, the catalytic activity was tested in four systems (US, NF, US/NF and NF/US/H_2_O_2_). The optimal conditions were fixed at 0.1 g/mL NF, initial 5.00 mg/L RhB, 300-W ultrasonic power, pH = 3 and 5.00 mg/L H_2_O_2_. The result of the dissolution of nickel showed that the concentration of nickel ion was measured as 2.50 mg/L. The removal efficiency of RhB decreased significantly with the increase of the quencher tertiary butanol due to quenching of the Fenton reaction. When the heterogeneous Fenton reaction is combined with TiO_2_-photocatalysis, the pollutant removal efficiency is enhanced significantly. Through this synergistic effect, 22% and 80% of acetochlor was degraded within 10 min and 80 min, respectively. The degradation efficiency reaches 98% at 100 min, which is high relative to the efficiencies reported for other methods in other conditions.
